# Detecting Sjögren’s disease from parotid gland ultrasound radiomics

**DOI:** 10.1093/rheumatology/keag409

**Published:** 2026-08-11

**Authors:** Gamze Akkuzu, Omer Faruk Durugol, Sena Tolu, Muhammed Furkan Dasdelen, Mehmet Karagulle, Kanullah Suleyman, Bilgin Karaalioğlu, Rabia Deniz, Duygu Sevinç Özgür, Fatih Yıldırım, Cemal Bes

**Affiliations:** Department of Rheumatology, Başakşehir Çam and Sakura City Hospital, Istanbul, Turkey; International School of Medicine, Istanbul Medipol University, Istanbul, Turkey; Faculty of Medicine, Department of Physical Medicine and Rehabilitation, Istanbul Medipol University, Istanbul, Turkey; International School of Medicine, Istanbul Medipol University, Istanbul, Turkey; Institute of AI for Health, Helmholtz Munich, Neuherberg, Germany; Department of Radiology, Başakşehir Çam and Sakura City Hospital, Istanbul, Turkey; Department of Radiology, Başakşehir Çam and Sakura City Hospital, Istanbul, Turkey; Department of Rheumatology, Başakşehir Çam and Sakura City Hospital, Istanbul, Turkey; Department of Rheumatology, Başakşehir Çam and Sakura City Hospital, Istanbul, Turkey; Department of Rheumatology, Başakşehir Çam and Sakura City Hospital, Istanbul, Turkey; Department of Rheumatology, Başakşehir Çam and Sakura City Hospital, Istanbul, Turkey; Department of Rheumatology, Başakşehir Çam and Sakura City Hospital, Istanbul, Turkey

**Keywords:** machine learning, ultrasonography, Sjögren’s disease

## Abstract

**Objectives:**

Salivary gland ultrasonography is a promising non-invasive modality for the evaluation of Sjögren’s disease (SjD), but its diagnostic utility is limited by operator dependency. This study aimed to evaluate the classification performance of radiomics-based machine learning using parotid gland ultrasonography and to compare it with conventional visual assessment.

**Methods:**

A total of 866 parotid gland ultrasound images from 202 participants were included: 123 patients fulfilling the 2016 ACR/EULAR criteria for SjD, 33 healthy controls, 24 non-Sjögren sicca patients and 22 incomplete SjD cases. A total of 104 radiomic features describing intensity, texture and micro-texture patterns were extracted. A 5-fold soft-voting support vector machine (SVM) ensemble was trained on confirmed SjD and healthy participants; non-Sjögren sicca and incomplete SjD cases were reserved for the held-out test set. SHapley Additive exPlanations (SHAP) analysis was used for model interpretability.

**Results:**

The SVM ensemble achieved an area under the receiver operating characteristic curve (AUC) of 0.99 for binary classification between SjD and healthy controls, with 0.94 accuracy, 0.86 sensitivity and 0.96 specificity, outperforming radiologist assessments (accuracy: 0.62 and 0.72). SHAP analysis identified intensity dispersion metrics, GLCM-based texture features and LBP micro-texture patterns as the strongest predictors. PCA and feature-level analyses demonstrated substantial overlap in radiomic features between non-Sjögren sicca and confirmed SjD patients.

**Conclusion:**

Radiomics-based machine learning demonstrated high classification performance for distinguishing SjD from healthy controls using parotid gland ultrasonography. Quantitative ultrasound analysis may serve as an objective adjunctive tool in SjD assessment, although validation in larger multicentre cohorts is required.

Rheumatology key messages

- Radiomics-based ultrasound accurately differentiates Sjögren’s disease from healthy controls using quantitative imaging features.
- Quantitative image analysis reveals subtle glandular abnormalities not readily captured by visual assessment.
- Quantitative radiomics may improve diagnostic standardization and support clinical decision-making in Sjögren’s disease.

## Introduction

Sjögren’s disease (SjD) is a chronic autoimmune disorder primarily characterized by sicca symptoms, including oral and ocular dryness, as well as fatigue, arthralgia and arthritis [1]. The 2016 American College of Rheumatology/European League Against Rheumatism (ACR/EULAR) classification criteria represent the most recently adopted criteria set for the classification of SjD. Within this framework, salivary gland histopathology and anti-SSA antibody positivity carry the greatest diagnostic weight, each assigned three points. The criteria further include objective assessments of glandular involvement, namely the Schirmer test and ocular staining score (OSS) for lacrimal gland evaluation, as well as unstimulated whole salivary flow (UWSF) for the assessment of salivary gland function [2].

Although salivary gland biopsy (SGB) plays a critical role in diagnosis, its invasive nature may lead to undesirable adverse effects such as pain, bleeding and nerve injury [3, 4], which may result in patient reluctance to undergo the procedure. In recent years, salivary gland ultrasonography (SGUS) has been increasingly used as a non-invasive, radiation-free, relatively easy-to-perform and repeatable modality in daily clinical practice [5]. Assessment of salivary gland morphology by SGUS is therefore considered a promising adjunctive tool that may reduce reliance on SGB in selected clinical scenarios.

However, SGUS evaluation is associated with several challenges, primarily due to the subjective nature of image interpretation and its operator dependency. Previous studies in SjD have demonstrated that the diagnostic performance of SGUS varies considerably according to examiner experience, resulting in heterogeneous sensitivity and specificity estimates [6, 7]. In this context, machine learning–based imaging approaches, particularly radiomics, may help overcome these limitations by enabling objective, quantitative and reproducible analysis of ultrasound images. Radiomic analysis of parotid gland ultrasonography may capture subtle textural and structural alterations that are not readily appreciable by conventional visual assessment alone.

Therefore, the primary aim of this study was to evaluate the classification performance of radiomics-based machine learning applied to parotid gland ultrasonography for distinguishing patients with confirmed SjD from healthy controls, and to identify radiomic features that reflect SjD-related parotid gland changes.

## Materials and methods

### Data collection

Between January 2023 and February 2024, we included all patients with a known diagnosis of SjD, as well as those referred to the Rheumatology Clinic of Başakşehir Çam and Sakura City Hospital for evaluation of sicca symptoms suggestive of SjD. Patients with coexisting autoimmune rheumatic diseases, a history of head and neck radiotherapy, or prior parotid gland surgery were excluded.

Demographic data were collected for all participants. A comprehensive clinical evaluation was performed, including the Schirmer test, labial salivary gland biopsy (LSGB), UWSF and anti-SSA antibody testing. OSS data were unavailable, as this assessment is not routinely performed at our institution. Therefore, patient classification was based on the available components of the 2016 ACR/EULAR criteria.

Patients fulfilling the available classification criteria were categorized as the SjD group. Patients who did not meet the classification criteria based on available clinical, serological and histopathological data were classified as the non-Sjögren sicca group. These patients were referred primarily because of sicca symptoms, but no objective serological or histopathological evidence supporting SjD was identified. This group consisted exclusively of patients negative for both anti-SSA antibodies and LSGB findings. Because these patients lacked both anti-SSA positivity and LSGB evidence, the absence of OSS data did not affect their classification status.

Given the absence of OSS data, individuals demonstrating partial objective SjD-related findings (anti-SSA positivity or LSGB focus score ≥1) but not fulfilling the available classification criteria were categorized as a separate ‘incomplete SjD’ subgroup for exploratory analyses.

A healthy control group, consisting of volunteers recruited from hospital staff and the general population, was also included. Controls had no history or clinical evidence of autoimmune disease or sicca symptoms, no chronic medical conditions, and were not using medications known to affect salivary gland function, such as antidepressants or antihistamines.

All ultrasound examinations were performed by an experienced sonographer with 10 years of expertise, using a high-resolution linear transducer (4–12 MHz; Philips Lumify L12-4 linear array transducer). With the patient in the supine position, the neck slightly extended and the face turned to the contralateral side, both parotid glands were examined in the longitudinal plane posterior to the mandibular ramus, and multiple longitudinal images of each parotid gland were recorded for subsequent analysis. The recorded images were subsequently annotated, and glandular regions were manually delineated by the same operator to minimize annotation variability and ensure consistency across all images. In total, we collected 866 images from 202 patients (4.29 ± 2.05 images per patient). Of these, 123 patients were diagnosed with SjD, 33 were healthy controls, 24 had non-Sjögren sicca and 22 had incomplete SjD.

Ultrasonographic images were independently evaluated by two radiologists with five years of experience, both blinded to clinical data and diagnosis. Visual assessment was performed using the recorded static ultrasonographic images based on overall parenchymal heterogeneity, hypoechoic areas, echogenic bands and distortion of glandular architecture. Each image was categorized into one of three groups: healthy, if the gland parenchyma appeared normal; possible SjD, if mild to moderate parenchymal changes were present; or SjD, if severe parenchymal changes were present.

This study was conducted in accordance with the Declaration of Helsinki. The study protocol was approved by the Clinical Research Ethics Committee of Istanbul Medipol University (Approval No: E-10840098–772.02–7777).

#### Radiomics features

Radiomics features are quantitative descriptors derived mathematically from medical images to capture tissue characteristics that may be imperceptible to the human eye. In this study, grayscale US images, with pixel intensities ranging from 0 (anechoic/black) to 255 (hyperechoic/white), were processed to extract a total of 104 radiomic features per image (Supplementary Table S1). The feature extraction pipeline was designed to capture the structural heterogeneity characteristic of SjD using the *PyRadiomics* library [8] for standard radiomics features and scikit-image library for local binary pattern and entropy features. In accordance with standard radiomic nomenclature, the 104 extracted features were categorized into three primary families:

##### Intensity-based statistics (*n* = 19)

These metrics quantify the global distribution of ultrasound echogenicity without considering spatial layout. This includes PyRadiomics first-order statistics (e.g. 90th Percentile, Robust Mean Absolute Deviation) and Shannon Entropy extracted via scikit-image, which measures the overall informational uncertainty of the intensity distribution.

##### Texture features (*n* = 75)

These second- and higher-order statistics quantify spatial heterogeneity and architectural disorganization. This family includes features derived from the Gray Level Co-occurrence Matrix (GLCM), Gray Level Dependence Matrix (GLDM), Gray Level Run Length Matrix (GLRLM), Gray Level Size Zone Matrix (GLSZM) and Neighbourhood Gray Tone Difference Matrix (NGTDM).

##### Micro-texture features (*n* = 10)

Evaluated using Local Binary Patterns (LBP) histograms derived via scikit-image, these features capture high-frequency local structural variations and uniform micro-patterns, offering robustness against baseline illumination changes.

In SjD, involvement within a single parotid gland may be heterogeneous, and the two parotid glands may be affected to different extents. Consequently, a single patient may demonstrate varying degrees of pathology across different US images of both parotid glands. To address this, we employed a maximum pooling (max-pooling) aggregation strategy. For each patient, all images were analyzed; however, the feature vector representing the patient was constructed by taking the maximum value of each feature across all images belonging to that patient. This approach is based on a worst-case scenario assumption, ensuring that if a region with high heterogeneity is present in any image, it decisively influences the diagnostic prediction.

### Machine learning based diagnostic

The total cohort of 202 participants was stratified into training (*N* = 99), validation (*N* = 25) and testing (*N* = 78) splits (Supplementary Table S2). To ensure a clean learning signal of the binary classification task, the training and validation sets consisted exclusively of confirmed SjD cases and healthy controls. Non-Sjögren sicca (*N* = 24) and incomplete SjD (*N* = 22) cases were excluded from model training and reserved exclusively for exploratory evaluation within the strictly held-out test set.

To map the radiomic feature space to a diagnostic output, we designed a supervised learning framework. Based on performance stability, the SVM (Support Vector Machine) was selected as the core classifier. SVMs operate by constructing an optimal hyperplane in a high-dimensional space to separate data classes with the maximum possible margin. The models were trained with the LibSVM implementation in scikit-learn, utilizing the Sequential Minimal Optimization (SMO) solver. Because standard SVMs output raw distance scores, internal Platt scaling was enabled to convert these scores into probabilities (0–1), facilitating accurate probability analysis. Feature standardization was integrated directly into the training pipeline using Z-score normalization.

Hyperparameter optimization was done using Grid Search. This optimization showed preference for regularization strength of C = 0.1 for the majority of folds, with the optimal decision boundary varying between Linear and Radial Basis Function (RBF) kernels (Supplementary Table S3). Class imbalance was addressed by setting class weights to ‘balanced’ across all training folds.

To ensure robust generalization and minimize the bias of any single data split, the final classification output was based on a 5-fold soft-voting ensemble [9]. The probability estimates from five independently trained SVM models were averaged to generate a final disease probability score for each patient.

### Feature importance analysis

Machine learning models are often black boxes, obscuring the biological rationale behind their predictions. To bridge the gap between algorithmic performance and clinical interpretability, we utilized SHapley Additive exPlanations (*SHAP*) [10].

SHAP is a game-theoretic approach that assigns each feature an importance value for a particular prediction. Unlike traditional feature importance methods (which only show magnitude), SHAP reveals directionality: it quantifies whether a high value of a feature (e.g. high Shannon Entropy) pushes the prediction towards SjD or healthy classification.

We implemented the KernelExplainer to approximate SHAP values across our heterogeneous ensemble. Global feature importance was derived by averaging the absolute SHAP values across the entire development dataset to find out which features affected the end diagnosis the most on average. This analysis allows us to validate the model’s logic against known pathophysiology.

### Evaluation metrics and statistical analysis

The primary metric for model evaluation was the Area Under the Receiver Operating Characteristic Curve (AUC-ROC), as it provides a threshold-independent measure of the model’s discriminative ability. We calculated a balanced threshold (Youden’s J [11]) based on the validation folds, optimizing for the best trade-off between sensitivity and specificity.

Standard metrics including precision, recall (sensitivity), specificity, negative predictive value (NPV), accuracy and F1 score were calculated. We report these metrics for the SVM model with the Youden’s J threshold (Supplementary Table S4).

After identifying the top features based on SHAP, we computed their mean and standard deviation in the hold-out test set and conducted statistical testing to assess group differences and effect sizes between SjD, healthy and non-Sjögren sicca patients. Since the data did not follow a normal distribution, we used the non-parametric Mann–Whitney *U* test for pairwise comparisons. To account for the multiple testing problem, a Bonferroni correction was applied. Statistical significance was defined as an adjusted *P*-value < 0.05.

The non-Sjögren sicca and incomplete SjD cohorts were maintained as a strictly held-out test set. These patients were passed through the trained ensemble to evaluate where their radiomic signatures positioned them relative to the clear healthy and SjD distributions, effectively testing the model’s ability to interpret borderline clinical presentations.

## Results

### Characteristic of participants

The participants’ demographic characteristics and the clinical components corresponding to the ACR/EULAR classification criteria are summarized in Table 1.

**Table 1:** Characteristics of participants.

Feature	SjD	Healthy	Non-Sjögren sicca	Incomplete SjD
Total patients	*N* = 123	*N* = 33	*N* = 24	*N* = 22
Age (mean ± s.d.)	47.3 ± 13.5	40.1 ± 7.8	42.6 ± 10.3	46.7 ± 13.3
Female (%)	98.4%	93.9%	91.7%	90.9%
Clinical components				
LSGB focus score ≥1	89/123 (72.4%)	N/A	0/24 (0.0%)	3/22 (13.6%)
Anti-SSA positive	103/123 (83.7%)	N/A	0/24 (0.0%)	19/22 (86.4%)
Schirmer’s test ≤ 5 mm/min	69/123 (56.1%)	N/A	13/24 (54.2%)	0/22 (0.0%)
UWSF ≤ 0.1 ml/min	60/123 (48.8%)	N/A	9/24 (37.5%)	0/22 (0.0%)
Oral Dryness Duration (s) (Median [Q1–Q3])	4.8 [1.0–6.0]	N/A	4.0 [1.0–5.2]	1.1 [0.0–2.0]
Ocular Dryness Duration (s) (Median [Q1–Q3])	5.6 [1.5–6.5]	N/A	3.9[1.0–5.0]	1.1 [0.0–2.0]
ACR/EULAR Score (Median [Q1–Q3])	6.0 [4.0–7.0]	N/A	1.0 [0.8–1.0]	3.0 [3.0–3.0]

The cohort was predominantly female across all subgroups (90.9–98.4%). The healthy control group was comparatively younger (40.1 ± 7.8 years) than the SjD group (47.3 ± 13.5 years) (*P* = 0.002). Within the SjD cohort, objective clinical markers were highly prevalent: 72.4% (89/123) demonstrated SGB positivity, and 83.7% (103/123) were positive for anti-SSA antibodies. In contrast, the non-Sjögren sicca cohort, despite exhibiting sicca symptoms (e.g. 54.2% positive Schirmer test), lacked serological and histopathological confirmation (0.0% biopsy or anti-SSA positivity), resulting in a substantially lower median ACR/EULAR score (1.0) compared with the SjD group (6.0).

### Radiomic features identifies Sjögren’s disease from parotid US images

**Figure 1:**
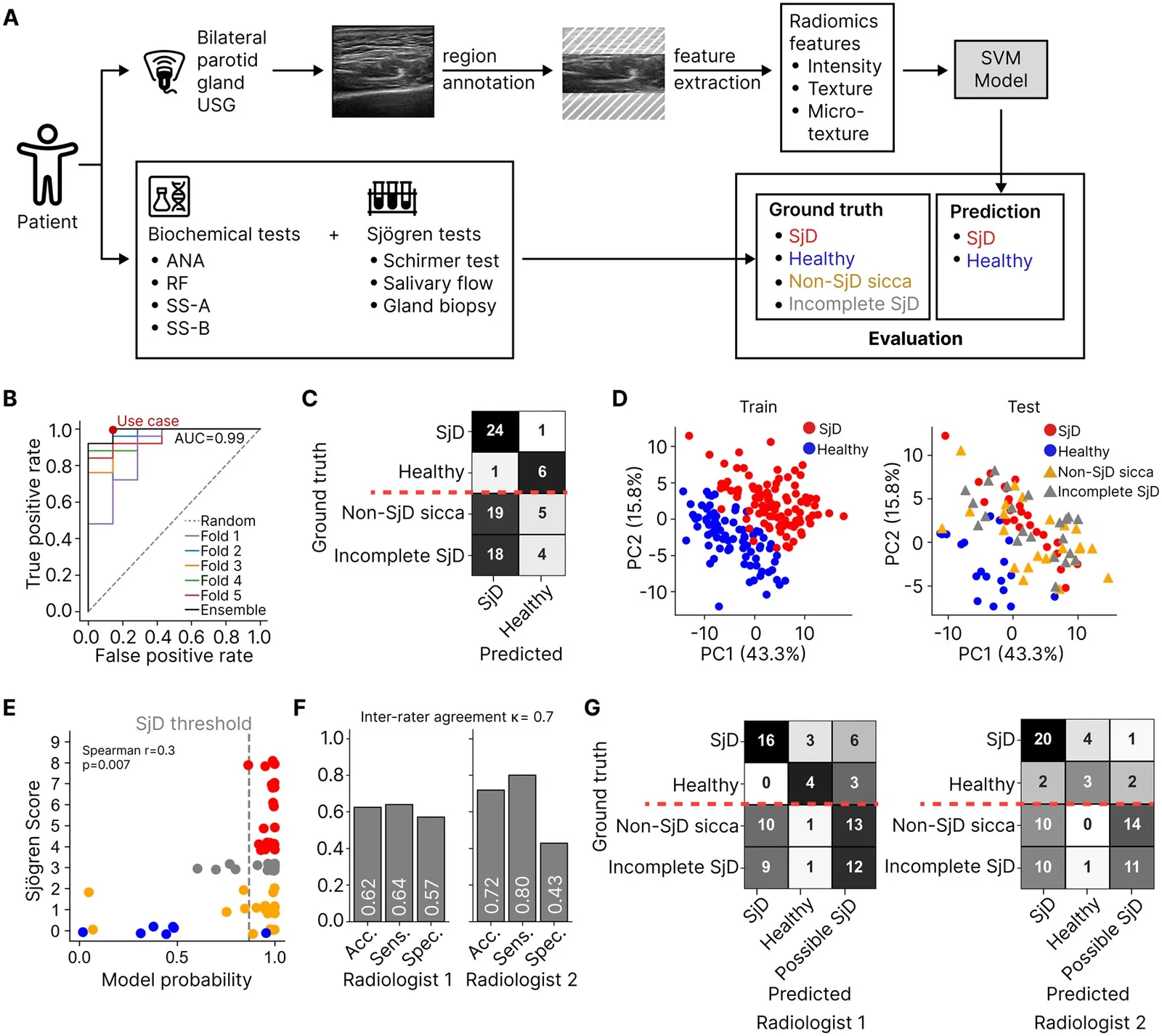
Radiomics features successfully predict Sjögren from US images. (**A**) Radiomics workflow extracts three key groups of features which the SVM model utilizes to predict the ground truth without knowing the lab test values at all. (**B**) Receiver Operating Characteristic (ROC) curve shows that the 5-fold ensemble is better than any individual fold’s performance on the test set. (**C**) Confusion matrix of the SVM model shows excellent performance. (**D**) PCA shows that both the train and test set are separable. In the test set, the non-Sjögren sicca and incomplete SjD closely follow the distribution of SjD. (**E**) AI predicted probability correlates with the composite ACR/EULAR clinical severity score (Spearman *r* = 0.3, *P* = 0.007). (**F**) Performance metrics of human radiologists are good and substantially consistent with 0.7 kappa, including the prediction of possible SjD class. (**G**) Confusion matrix of radiologist predictions

The overall workflow is summarized in Fig. 1A. Bilateral parotid gland ultrasound images were manually annotated to define the glandular region of interest, from which 104 intensity, texture and micro-texture features were extracted. These features were max-pooled across images for each participant and supplied to the SVM ensemble. Clinical, serological and histopathological assessments used to establish the ground-truth diagnosis.

The 5-fold SVM ensemble successfully discriminated SjD from healthy controls, achieving an AUC-ROC of 0.99 on the independent test set (Fig. 1B). The ensemble approach mitigated the variance of individual data splits, yielding a robust diagnostic performance with 0.94 Accuracy, 0.86 Sensitivity and 0.96 Specificity (Fig. 1C).

Beyond binary classification, the radiomics feature space provided additional insight into the clinical spectrum of the disease. PCA demonstrated that healthy and SjD cohorts formed distinct, linearly separable clusters. Non-Sjögren sicca and incomplete SjD patients showed a heterogeneous distribution, with some individuals positioned in an intermediate zone between the healthy and SjD clusters and others falling closer to the SjD cluster (Fig. 1D).

Furthermore, the model’s output functioned as a continuous indicator of SjD likelihood rather than a simple binary flag. The model probability of having SjD showed a modest positive correlation with the composite ACR/EULAR classification score (Spearman’s *r* = 0.3, *P* = 0.007) (Fig. 1E). All cases with SjD had probability higher than the 0.87 threshold [threshold determined in the use case (Fig. 1B)], except a single SjD case with probability 0.86. Incomplete SjD patients varied between 0.6–1.0 with 18/22 above the 0.87 threshold. The majority of non-Sjögren sicca patients (19/24) had a probability higher than 0.87. Three patients had lower than 0.1 probability, two non-Sjögren sicca and one healthy control.

The same test set was independently evaluated by two radiologists who were double-blinded and had no access to clinical information. This design was intentionally implemented to enable comparison based solely on imaging features. Radiologist 1 achieved an accuracy of 0.62 for diagnosing SjD from US images, with a sensitivity of 0.64 and specificity of 0.57. Radiologist 2 achieved an accuracy of 0.72, with a sensitivity of 0.80 and specificity of 0.43. Inter-rater agreement was substantial (Cohen’s κ = 0.70). The relatively lower performance of human readers may reflect the absence of clinical context and real-time dynamic examination, both of which are typically incorporated into routine diagnostic decision-making.

### Feature importance analysis

**Figure 2:**
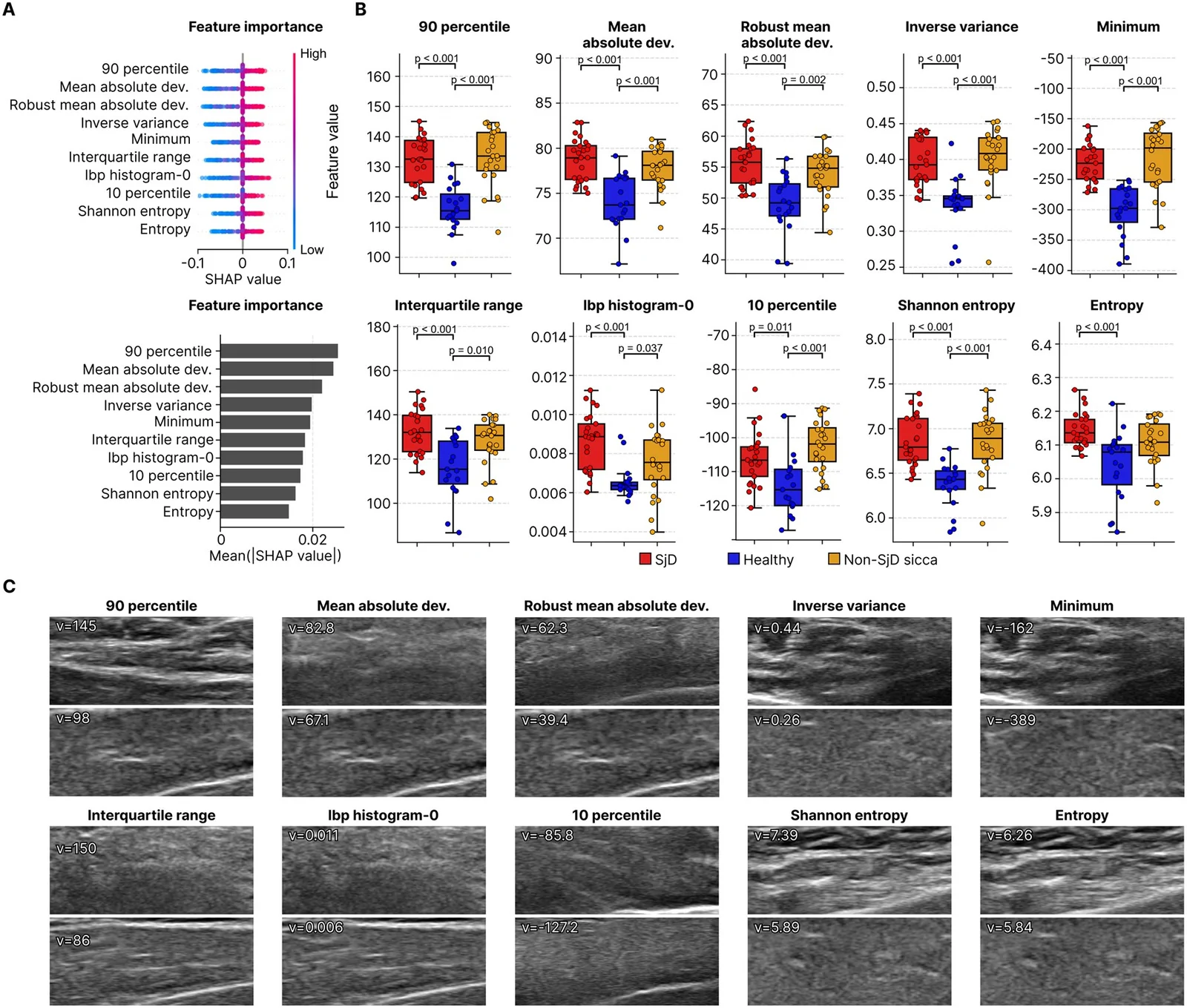
Radiomic features help distinguish Sjögren patients from healthy controls. (**A**) SHAP analysis. Top: Beeswarm plot showing the impact of feature values on model output; each dot represents a patient. Red dots (high feature value) on the positive SHAP side indicate that the feature pushes the prediction towards SjD. Bottom: Global feature importance ranking based on mean absolute SHAP values. (**B**) Distribution of top features across clinical groups. Non-Sjögren sicca patients exhibit feature values significantly different from healthy controls for most of the important features, similar to the SjD group. SjD patients are statistically distinct from healthy controls. (**C**) US image gallery: Top row shows SjD glands with higher heterogeneity and textural coarseness, while bottom row shows healthy glands with higher homogeneity and textural smoothness

SHAP analysis revealed that features from all three radiomic families contributed to the diagnostic prediction, with all top features showing higher values in SjD glands than healthy glands (Fig. 2A).

Among intensity-based statistics, the 90th percentile, 10th percentile, minimum, interquartile range, mean absolute deviation, robust mean absolute deviation, entropy and Shannon entropy were highly predictive.

The 90th percentile represents the intensity value below which 90% of pixels fall, while the 10th percentile represents the value below which 10% of pixels fall. Higher values of the 90th percentile in SjD glands indicate that the upper tail of the intensity distribution is shifted towards brighter pixels, while lower values of the 10th percentile and minimum indicate that the lower tail is shifted towards darker pixels. Together, these findings demonstrate that SjD glands exhibit a widened intensity range compared with healthy tissue, with both brighter and darker pixel populations present within the same gland. This pattern is mathematically consistent with the coexistence of hyperechoic and hypoechoic regions, a qualitative feature of parenchymal inhomogeneity described in SjD [12].

The interquartile range, mean absolute deviation and robust mean absolute deviation are measures of statistical dispersion. The interquartile range quantifies the spread of the central 50% of the intensity distribution, capturing the range between the 25th and 75th percentiles. Mean absolute deviation calculates the average absolute distance of all intensity values from the mean, providing a global measure of dispersion, whereas robust mean absolute deviation performs the same calculation but excludes pixels below the 10th and above the 90th percentile, making it less sensitive to outliers. Higher values of all three dispersion metrics in SjD glands indicate greater variability in pixel intensities, reflecting increased heterogeneity of the glandular parenchyma. This heterogeneity likely arises from the alternating areas of tissue damage and inflammation that characterize the disease [13].

Entropy and Shannon entropy measure the randomness or irregularity of the intensity distribution, with higher values indicating greater unpredictability in pixel values. While PyRadiomics entropy divides the pixels into bins first, Shannon entropy calculates them pixel-wise. Increased entropies in SjD glands reflect loss of the uniform, organized architecture seen in healthy parotid tissue and its replacement by a more disorganized parenchymal pattern.

Among texture features, inverse variance from the GLCM was particularly influential. Inverse variance measures local homogeneity (in contrast to global) with a quadratic weighting of pixel differences, where higher values indicate greater similarity between neighbouring pixels. Higher values in SjD glands indicate that within distinct regions, whether hyperechoic fibrotic areas or hypoechoic lymphocytic infiltrates, the parenchyma is locally uniform even as the coexistence of these regions increases global dispersion.

Among micro-texture features, LBP histogram-0 was a key predictor. LBP histogram-0 specifically measures the frequency of uniform micro-patterns in the image. Higher values in SjD glands indicate alterations in fine-grained textural details, suggesting disruption of the normal micro-architecture of the glandular parenchyma.

To strictly compare borderline non-Sjögren sicca cases against the clear-cut groups, we excluded incomplete SjD patients from the statistical significance analysis of top 10 important features stratified by disease status (Fig. 2B). SjD patients exhibited significantly higher statistics compared with healthy controls (*P* < 0.001 for all important features, except *P* = 0.011 for 10 percentile). Similarly, non-Sjögren sicca patients exhibited feature values significantly distinct from the healthy baseline (*P* < 0.05 for all important features, except *P* > 0.05 for entropy), often aligning closely with the SjD distribution. Visual inspection of representative parotid images confirms the general difference between healthy and SjD findings: healthy glands display smooth, uniform textures; whereas high-probability SjD glands show coarse, heterogeneous textures (Fig. 2C).

## Discussion

In this study, we developed and validated a machine learning pipeline based on ultrasound radiomics for the objective classification of SjD. Our 5-fold SVM ensemble achieved high diagnostic performance (AUC 0.99) on an independent test set, demonstrating that quantitative analysis of parotid gland texture can reliably distinguish SjD from healthy controls.

The concept of quantitative image analysis of the parotid gland in SjD is not new, but prior efforts have been limited in scope and methodology. Chikui *et al.* [14] were among the first to apply quantitative texture analysis to parotid gland ultrasonography in Sjögren patients, demonstrating that fractal-based Hurst coefficients could differentiate the Sjögren group from non-Sjögren patients and that these indices correlated with the sialographic stage of glandular destruction. Critically, that study found that conventional first-order statistics such as mean grey level and standard deviation failed to achieve significant discrimination between groups, whereas higher-order spatial features did–a finding that is directly consistent with our findings. Our SHAP analysis confirms that intensity dispersion metrics (interquartile range, robust mean absolute deviation) and spatial heterogeneity features (GLCM inverse variance, LBP micro-texture patterns) are the strongest predictors, underscoring that it is the spatial organization of echogenicity–not its absolute level–that encodes the pathological signature of SjD. Our work extends Chikui *et al.*'s foundational insight by deploying a far richer feature set (104 radiomic features spanning first-order, texture and micro-texture families), a modern supervised ensemble classifier with probabilistic output, and a substantially larger and clinically well-characterized cohort that includes not only confirmed and healthy groups but also diagnostically ambiguous patient categories.

In parallel with these early texture analysis efforts, the field has increasingly turned to deep learning as a means of automating salivary gland assessment. Kise *et al.* [15] applied a convolutional neural network to parotid and submandibular gland ultrasound images, achieving an AUC of 0.948 for the parotid gland in distinguishing SjD from non-SjD patients with dry mouth, and demonstrated that this significantly outperformed inexperienced radiologists (AUC 0.810). Our results are consistent with this trajectory: our radiomics SVM ensemble similarly surpasses human observers, and the interobserver variability observed in our study (κ = 0.70) parallels the variability in human evaluation documented across prior studies.

More recently, Niu *et al.* [16] established a ResNet-50 deep learning model evaluated across submandibular, parotid and lacrimal glands in a multicentre dataset, achieving AUCs of 0.92, 0.93 and 0.91 respectively in the model cohort, and outperforming two radiologists in all gland regions. Their study represents a significant advance in multi-glandular AI-assisted assessment of SjD and highlights the potential of deep learning to democratize expert-level imaging evaluation even in resource-limited settings. Our study is complementary with this approach. Where Niu *et al.* focused on end-to-end deep learning classification, our radiomics pipeline explicitly decomposes diagnostic signal into interpretable, biologically grounded features. Through SHAP analysis, we demonstrate which specific quantitative properties–parenchymal intensity spread, textural dispersion, local micro-architectural disruption–drive classification, thereby offering mechanistic insight that black-box deep learning models do not readily provide.

The radiomics approach of Vukicevic *et al.* [17] which provided the conceptual basis for applying handcrafted quantitative features to SGUS images in SjD, focused primarily on gland segmentation and radiomic scoring relative to the De Vita visual scoring system. Our work differs in that we train and validate against the 2016 ACR/EULAR classification criteria as the reference standard and include a healthy control group, making our diagnostic model directly applicable to the clinical framework currently in use for binary SjD classification.

Notably, non-Sjögren sicca patients received high disease probability and exhibited radiomic feature distributions largely similar to those of confirmed SjD patients (Fig. 2B). While this finding cannot be interpreted as evidence of early or subclinical SjD in the absence of longitudinal follow-up data, it carries an important clinical implication: parotid gland parenchymal texture alterations detectable by radiomics are not exclusive to serologically or histopathologically confirmed SjD. This suggests that radiomic features capture a broader signature of glandular involvement that may be shared across sicca conditions of different aetiologies. The high disease probability observed in incomplete SjD patients–who carry partial serological or histopathological SjD evidence but do not yet fulfil the full classification criteria–raises the possibility that radiomic biomarkers may detect subclinical glandular pathology that precedes formal diagnostic confirmation.

In conclusion, our radiomics pipeline provides an objective and quantitative framework for ultrasound-based SjD classification. While the model demonstrated high performance in distinguishing confirmed SjD from healthy controls, further validation in larger and clinically heterogeneous cohorts is required before broader diagnostic application.

### Limitations

Several limitations of this study should be acknowledged. First, the cohort was recruited from a single tertiary centre using a single ultrasonography device, which may limit generalizability. Because radiomic features are sensitive to acquisition settings, external validation should be conducted. The healthy control group was somewhat younger than the patient groups, though age is unlikely to fully account for the magnitude of radiomic differences observed.

Second, since OSS assessment was not routinely performed at our institution, the classification of non-Sjögren sicca and incomplete SjD feature groups relied more heavily on the remaining components of the 2016 ACR/EULAR criteria. Consequently, some patients with predominantly ocular manifestations may not have been fully captured within the classification framework used in this study.

Third, the study relied on standardized static ultrasonographic images rather than dynamic real-time assessment. Routine SGUS evaluation in clinical practice incorporates dynamic multiplanar examination together with clinical context, both of which may influence human interpretation. Therefore, the radiologist performance observed in this study may not fully reflect routine clinical diagnostic conditions.

Finally, the feature extraction remains dependent on the initial region of interest (ROI) selection. Future work should focus on feature extraction combined with fully automated segmentation and multicentre validation to ensure generalizability across different ultrasound device manufacturers.

## Supplementary Material

keag409_Supplementary_Data

## Data Availability

The data collected and used in this study is available upon reasonable request and appropriate ethics statement.
