# Supplementary material for: Detecting Sjögren’s disease from parotid gland ultrasound radiomics
Source: Rheumatology (Oxford). 2026 Aug 11;65(8):keag409. doi: 10.1093/rheumatology/keag409 (PMC13509949; doi:10.1093/rheumatology/keag409)
Supplement: Supplementary file 1 [file keag409_supplementary_data.docx]

# Supplementary Findings

**Supplementary Table S1: Our pipeline extracts 104 interpretable morphological features of Sjögren US images.** The table provides a list of features for three feature groups. For each group we provide the implementation source of feature extraction in the *Extracted with* column. Detailed descriptions of every feature and their computation formulas are provided in the documentation of the source libraries. Column *Feature prefix* gives the prefix used for each feature group in the tables produced after feature extraction.

| **Feature group** | **Feature prefix** | **Extracted with** | **Feature names** |
| --- | --- | --- | --- |
| Intensity-based statistical features | firstorder | pyradiomics | 10 Percentile |
|  |  |  | 90 Percentile |
|  |  |  | Energy |
|  |  |  | Entropy |
|  |  |  | Interquartile Range |
|  |  |  | Kurtosis |
|  |  |  | Maximum |
|  |  |  | Mean Absolute Deviation |
|  |  |  | Mean |
|  |  |  | Median |
|  |  |  | Minimum |
|  |  |  | Range |
|  |  |  | Robust Mean Absolute Deviation |
|  |  |  | Root Mean Squared |
|  |  |  | Skewness |
|  |  |  | Total Energy |
|  |  |  | Uniformity |
|  |  |  | Variance |
|  | entropy | skimage | Shannon Entropy |
| Texture features | glcm | pyradiomics | Autocorrelation |
|  |  |  | Cluster Prominence |
|  |  |  | Cluster Shade |
|  |  |  | Cluster Tendency |
|  |  |  | Contrast |
|  |  |  | Correlation |
|  |  |  | Difference Average |
|  |  |  | Difference Entropy |
|  |  |  | Difference Variance |
|  |  |  | Inverse Difference |
|  |  |  | Inverse Difference Moment |
|  |  |  | IDM Normalized |
|  |  |  | ID Normalized |
|  |  |  | Informational Measure of Correlation 1 |
|  |  |  | Informational Measure of Correlation 2 |
|  |  |  | Inverse Variance |
|  |  |  | Joint Average |
|  |  |  | Joint Energy |
|  |  |  | Joint Entropy |
|  |  |  | Maximal Correlation Coefficient |
|  |  |  | Maximum Probability |
|  |  |  | Sum Average |
|  |  |  | Sum Entropy |
|  |  |  | Sum Squares |
|  | gldm | pyradiomics | Dependence Entropy |
|  |  |  | Dependence Non Uniformity |
|  |  |  | Dependence Non Uniformity Normalized |
|  |  |  | Dependence Variance |
|  |  |  | Gray Level Non Uniformity |
|  |  |  | Gray Level Variance |
|  |  |  | High Gray Level Emphasis |
|  |  |  | Large Dependence Emphasis |
|  |  |  | Large Dependence High Gray Level Emphasis |
|  |  |  | Large Dependence Low Gray Level Emphasis |
|  |  |  | Low Gray Level Emphasis |
|  |  |  | Small Dependence Emphasis |
|  |  |  | Small Dependence High Gray Level Emphasis |
|  |  |  | Small Dependence Low Gray Level Emphasis |
|  | glrlm | pyradiomics | Gray Level Non Uniformity |
|  |  |  | Gray Level Non Uniformity Normalized |
|  |  |  | Gray Level Variance |
|  |  |  | High Gray Level Run Emphasis |
|  |  |  | Long Run Emphasis |
|  |  |  | Long Run High Gray Level Emphasis |
|  |  |  | Long Run Low Gray Level Emphasis |
|  |  |  | Low Gray Level Run Emphasis |
|  |  |  | Run Entropy |
|  |  |  | Run Length Non Uniformity |
|  |  |  | Run Length Non Uniformity Normalized |
|  |  |  | Run Percentage |
|  |  |  | Run Variance |
|  |  |  | Short Run Emphasis |
|  |  |  | Short Run High Gray Level Emphasis |
|  |  |  | Short Run Low Gray Level Emphasis |
|  | glszm | pyradiomics | Gray Level Non Uniformity |
|  |  |  | Gray Level Non Uniformity Normalized |
|  |  |  | Gray Level Variance |
|  |  |  | High Gray Level Zone Emphasis |
|  |  |  | Large Area Emphasis |
|  |  |  | Large Area High Gray Level Emphasis |
|  |  |  | Large Area Low Gray Level Emphasis |
|  |  |  | Low Gray Level Zone Emphasis |
|  |  |  | Size Zone Non Uniformity |
|  |  |  | Size Zone Non Uniformity Normalized |
|  |  |  | Small Area Emphasis |
|  |  |  | Small Area High Gray Level Emphasis |
|  |  |  | Small Area Low Gray Level Emphasis |
|  |  |  | Zone Entropy |
|  |  |  | Zone Percentage |
|  |  |  | Zone Variance |
|  | ngtdm | pyradiomics | Busyness |
|  |  |  | Coarseness |
|  |  |  | Complexity |
|  |  |  | Contrast |
|  |  |  | Strength |
| Micro-texture features | lbp_hist | skimage | LBP Histogram Bin 0 |
|  |  |  | LBP Histogram Bin 1 |
|  |  |  | LBP Histogram Bin 2 |
|  |  |  | LBP Histogram Bin 3 |
|  |  |  | LBP Histogram Bin 4 |
|  |  |  | LBP Histogram Bin 5 |
|  |  |  | LBP Histogram Bin 6 |
|  |  |  | LBP Histogram Bin 7 |
|  |  |  | LBP Histogram Bin 8 |
|  |  |  | LBP Histogram Bin 9 |

**Supplementary Table S2: Patient splits**.The mean number of ultrasound images per patient varied across diagnostic groups: 4.71±1.82 for the diseased group, 3.06±1.92 for the healthy group, 3.58±2.06 for the suspicious group, and 4.55±2.44 for the undetermined group. The overall average for the entire cohort was 4.29±2.05.

|  | **Training** | | **Validation** | | **Test** | | **Total** | |
| --- | --- | --- | --- | --- | --- | --- | --- | --- |
| Number of: | Patients | Images | Patients | Images | Patients | Images | Patients | Images |
| **Diseased** | 79 | 366 | 19 | 94 | 25 | 119 | 123 | 579 |
| **Healthy** | 20 | 57 | 6 | 25 | 7 | 19 | 33 | 101 |
| **Suspicious** | 0 | 0 | 0 | 0 | 24 | 86 | 24 | 86 |
| **Undetermined** | 0 | 0 | 0 | 0 | 22 | 100 | 22 | 100 |

**Supplementary Table S3: Hyperparameters for the 5-fold SVM model**

| **Fold** | **Kernel** | **C (Regularization)** | **Gamma** | **Class Weight** |
| --- | --- | --- | --- | --- |
| 1 | Polynomial | 10.0 | 0.01 | Balanced |
| 2 | Linear | 0.1 | Scale | Balanced |
| 3 | RBF | 0.1 | Scale | Balanced |
| 4 | RBF | 0.1 | Scale | Balanced |
| 5 | Linear | 0.1 | Scale | Balanced |

**Supplementary Table S4: Performance metrics of the SVM model at Youden’s J threshold.**

| Precision | Recall | Specificity | NPV | Accuracy | F1 | AUC ROC | AUC PR |
| --- | --- | --- | --- | --- | --- | --- | --- |
| 0.96 | 0.96 | 0.86 | 0.86 | 0.94 | 0.96 | 0.97 | 0.99 |

**Supplementary Table S5: SVM outperformed other classic ML models.** To compare different models on the tabular feature-extracted radiomics data, we evaluated five supervised machine learning algorithms (Table S0): Multi-Layer Perceptron (MLP), Decision Trees (DT), Logistic Regression (LR), k-Nearest Neighbors (KNN), and Support Vector Machines (SVM). Precision-recall area under the curve (PR AUC) and receiver operating characteristics area under the curve (ROC AUC) were considered as the ranking metric for their threshold independent robustness.

| Model | Accuracy | Sensitivity | Specificity | F1 | Precision | NPV | ROC AUC | PR AUC |
| --- | --- | --- | --- | --- | --- | --- | --- | --- |
| SVM | **0.94** | 0.96 | **0.86** | **0.96** | **0.96** | 0.86 | **0.97** | **0.99** |
| MLP | 0.94 | **1.00** | 0.71 | **0.96** | 0.93 | **1.00** | 0.95 | **0.99** |
| LR | 0.88 | 0.92 | 0.71 | 0.92 | 0.92 | 0.71 | 0.93 | 0.95 |
| DT | 0.84 | 0.84 | **0.86** | 0.89 | 0.95 | 0.60 | 0.90 | 0.97 |
| KNN | 0.75 | 0.72 | **0.86** | 0.82 | 0.95 | 0.46 | 0.89 | 0.94 |
